# Simulation-Based Outreach Program Improves Rural Hospitals’ Team Confidence in Neonatal Resuscitation

**DOI:** 10.7759/cureus.28670

**Published:** 2022-09-01

**Authors:** Allison Zanno, Misty Melendi, Anya Cutler, Benjamin Stone, Micheline Chipman, Jeffrey Holmes, Alexa Craig

**Affiliations:** 1 Neonatology, Maine Medical Center, Portland, USA; 2 Center for Outcomes Research and Evaluation, Maine Medical Center, Portland, USA; 3 School of Medicine, Tufts University School of Medicine, Boston, USA; 4 Medical Education and Simulation, Maine Medical Center, Portland, USA; 5 Emergency Medicine, Maine Medical Center, Portland, USA; 6 Neonatal and Pediatric Neurology, Maine Medical Center, Portland, USA

**Keywords:** rural hospital, confidence, simulation, delivery room, resuscitation, neonatal

## Abstract

Introduction: Neonatal resuscitation is a high acuity, low occurrence event (HALO), and in rural community hospitals, low birth rates prevent providers from regular opportunities to maintain essential resuscitation skills. Simulation is an effective training modality for medical education, although resources for simulation are often limited in rural hospitals. Our primary objective was to test the hypothesis that in situ neonatal resuscitation simulation training improves rural hospitals' delivery room team confidence in performing key Neonatal Resuscitation Program^®^ (NRP^®^) skills. Our secondary objective was to compare confidence to performance as measured by adherence to NRP® guidelines.

Methods: We conducted a quasi-experimental pre-training survey and post-training survey of delivery room team confidence in NRP^®^ skills at five level one delivery hospitals before and after an in situ simulation training program. Participants included rural hospitals’ usual delivery room team members. Participants rated their confidence on a five-point Likert scale. Simulations were analyzed using an adapted version of a validated scoring tool for NRP^®^ adherence and presented as overall percentage scores.

Results: Our data demonstrate a significant improvement in self-assessed confidence levels pre- and post-simulation training in key areas of neonatal resuscitation. Participants reported higher confidence in airway management (4 vs. 3, p=0.003), emergency intravenous access (3 vs. 2, p=0.007), and the ability to manage a code in the delivery room (4 vs. 3, p=0.013) and the operating room (4 vs. 3, p=0.028). Improvements were also noted in their team member’s knowledge and skills to perform neonatal resuscitation. While improvements were appreciated in confidence, the performance of skills (NRP^®^ adherence scores) was often in the sub-optimal performance range.

Conclusions: An in situ-based neonatal resuscitation outreach simulation program improves self-confidence among rural delivery room teams. Additional research is needed to understand how to translate improved confidence into actual improved performance.

## Introduction

The Neonatal Resuscitation Program® (NRP®), created in 1987, is a standardized training program that equips healthcare teams that attend births with an evidence-based method of supporting neonates through the transition to the extrauterine environment [1]. While most neonates are successful in transitioning with minimal intervention, approximately five percent require positive pressure ventilation and two percent require additional measures including intubation [2]. Neonatal resuscitation, therefore, represents a high-acuity, low occurrence event (HALO), where a rapid skillful response by a delivery room team is pivotal in the reduction of neonatal morbidity and mortality [3]. However, in small hospitals, particularly in rural areas, low delivery rates lead to infrequent opportunities to implement NRP®. For example, in the state of Maine, one of the most rural states in the United States, over two-thirds of hospitals deliver less than one neonate per day. As such, it can be challenging for delivery room teams to regularly practice and maintain these essential NRP® skills, potentially leading to lower confidence [4] and potential competency.

Simulation training is widely used in a variety of medical disciplines to improve care, including in neonates [5-9]; and has been shown to improve communication [10], knowledge acquisition, and retention, and decrease mortality [11]. Consensus statements from the International Liaison Committee on Resuscitation (ILCOR) and the American Heart Association (AHA) have consistently recommended frequent simulation sessions to optimize skill retention [12] due to skill decline over time. We conducted in situ neonatal resuscitation simulation training for delivery room teams in five rural community hospitals through skills demonstration and simulated resuscitation scenarios. The goal of this study was to assess the impact of simulation training on delivery room team confidence in performing key NRP® skills as well as compare confidence to the performance in simulated resuscitation scenarios, as measured by adherence to NRP® guidelines. This article was previously presented as a meeting abstract at the 2022 IMSH Society for Simulation in Healthcare Annual Conference in Los Angeles, California, on January 16, 2022.

## Materials and methods

Study design

Quasi-experimental pre- and post-training surveys of delivery room team confidence in NRP® skills and teamwork were conducted at five level one delivery hospitals in Maine with less than 500 deliveries per year.

Participants

Our simulation team from our regional Level IV Neonatal Intensive Care Unit (NICU) including neonatologists, nurse practitioners, NICU nurses, NICU respiratory therapists, and simulation specialists created and ran this outreach neonatal education program. We invited nurses, respiratory therapists, advanced practice providers (APPs) such as midwives, and physicians to participate as this group reflects the rural hospital’s usual delivery room team. Participants were excluded if they did not complete NRP® in the two years prior to the simulation event as this is a requirement for employment. Before the simulation training event, participants reviewed learning material, including evidence-based neonatal resuscitation and teamwork articles and a video of neonatal resuscitation following NRP guidelines. At four hospitals, training was offered in two sessions, morning and afternoon, to accommodate the entire delivery room team staff, larger numbers of learners, and other clinical responsibilities. Consent was obtained for participation and video recording of simulations.

Confidence self-assessment

Participants were asked to complete a survey assessing confidence in neonatal resuscitation, which was sent via email at least two weeks prior to the event as well as in subsequent reminder emails. The survey was designed by the investigator team who are experts in neonatal resuscitation. Questions were created based on standard NRP resuscitations and common issues in taking care of neonates in rural hospitals (i.e., intravenous access). Delivery room team members rated their confidence in several components of neonatal resuscitation on a five-point Likert scale (1=no confidence/unprepared and 5=complete confidence/fully prepared). Identical post-training confidence surveys were administered on-site following training. These assessments were anonymous. This study was IRB approved with exempt status.

Simulation training

On the day of the simulation training, the delivery room team participated in timed specific skills training sessions: positive pressure ventilation, airway management, including laryngeal mask airway and intubation, emergency umbilical vein vascular access, medication administration, and needle decompression for pneumothorax. These skills were taught by a team of simulation-trained neonatologists, a neonatal nurse practitioner, a neonatal nurse, and a respiratory therapist all from the regional level IV NICU. The simulation nurse educator and technology expert conducted a simulation pre-brief before the simulation session started to orient learners to the high-fidelity newborn mannequin (Gaumard SUPER TORY® S2220 Advanced Newborn Patient Simulator; Gaumard Scientific, Miami, USA). 

The participants were then divided into three groups at random based on their clinical roles such that each team had at a minimum one general pediatrician, one respiratory therapist, and two nurses representing their native resuscitation team make-up. Each team then participated in three high-fidelity simulation scenarios: optimal airway management, including intubation, pneumothorax requiring needle decompression, and a full code requiring intubation, umbilical venous catheter placement, epinephrine, and volume administration. These simulation sessions were conducted in the delivery room located in the labor and delivery unit of the rural, community hospital using the participating hospital’s equipment. The simulations were live-streamed using audio-visual cameras set up by the simulation team and SimCapture (Laerdal Medical, Stavanger, Norway), for the other interprofessional team participants to view in an adjacent room at all sites. Debriefing sessions were conducted after each simulation and were led by the simulation-trained multi-disciplinary team using open-ended questions that had the participants explore medical knowledge, teamwork, communication, latent safety threats, and specific system challenges at their local hospitals.

NRP® adherence

The airway management and full code simulations were recorded using SimCapture and cameras and stored on a firewall-protected server for subsequent review by the research team. These were analyzed using an adapted version of the validated scoring tool for adherence to NRP® originally created by van der Heide et al. [13]. This tool divides NRP® adherence into eight categories, such as positive pressure ventilation and intubation, for which scores are assigned based on the appropriateness and execution of the skill (0 not done, 1 done poorly or not on time, and 2 done correctly). The sub-scores are weighted and combined to generate an overall percentage score ranging from 0-100%. For example, performing the correct positive pressure ventilation technique counts more towards the overall score than checking the heart rate. All simulation recordings were jointly scored for NRP® adherence by a team of two simulation-trained neonatologists.

Analysis

To analyze significant changes in confidence before and after training sessions, we treated Likert-scale questions as ordinal variables and used a Cochran-Armitage trend test to assess statistical differences between the pre-and post-training survey answers. Because surveys were anonymous, the pre-and post-surveys had to be treated as independent groups rather than paired. NRP® adherence data for the full code scenario is presented as overall percentage scores (averaged between the morning and afternoon groups at each hospital). Overall NRP® adherence scores for each hospital were compared to pre-event confidence survey responses using violin plots. 

## Results

Simulation training occurred during 2018-2021 at five rural hospitals with delays due to the inability to meet in person for simulation during the beginning of the COVID-19 pandemic. A total of 123 participants (103 females and 20 males): 36 physicians, six midwives, 56 nurses, and 25 respiratory therapists participated in the in situ sessions. Hundred and one participants filled out the confidence survey (82%), including physicians (25 pre and 21 post), nurses (39 pre and 28 post), respiratory therapists (23 pre and 13 post), APPs (10 pre and two post), and those self-identified as other (four pre and four post). Participants varied in the amount of time in their current position, the number of deliveries attended, and the number of resuscitations attended in the last two years (Table 1).

**Table 1:** Participant demographics. ^1 ^Median (Minimum, Maximum) APP = Advanced Practice Provider; MD = Medical Doctor; DO = Doctor of Osteopathic Medicine; RT = Respiratory Therapist

Characteristic	APP, N=10^1^	MD/DO, N=25^1^	Other, N=4^1^	Nurse, N=39^1^	RT, N=23^1^
Number of years in current role	10 (2,19)	14 (0,33)	6 (4,20)	15 (0,40)	28 (5,45)
Number of deliveries in last 6 months	20 (5,50)	15 (0,40)	20 (0,40)	10 (0,40)	0 (0,40)
Unknown	0	0	0	1	0
Number of resuscitations in last 6 months	0 (0,20)	5 (0,25)	3 (0,50)	2 (0,15)	2 (0,40)
Unknown	0	0	0	0	1

Self-assessed confidence levels demonstrate significant improvement comparing pre- to post-simulation training in multiple key areas of neonatal resuscitation (Table 2). Post in situ training scores reflected greater confidence in the following areas: airway management (4 vs. 3, p=0.003), emergency intravenous access (3 vs. 2, p=0.007), and ability to manage a code in the delivery room (4 vs. 3, p=0.013) and the operating room (4 vs. 3, p=0.028). In addition, improvements were noted in the knowledge of NRP®, team members’ knowledge, and skills to perform neonatal resuscitation. In a sub-analysis by delivery room team role, RNs and RRTs had the greatest confidence improvement (Table 2), whereas the physicians did not have a significant difference in confidence levels.

**Table 2:** Confidence scores presented overall and by participant role. Data was analyzed using a Cochrane-Armitage trend test. ^1^Median (minimum, maximum) ^*^ Indicates statistical significance by Cochran Armitage Test at p<0.05

Role	ALL		Nurses		Respiratory Therapist		Physicians	
Survey question	Pre, N = 101^1^	Post, N = 68^1^	Pre, N = 39^1^	Post, N = 28^1^	Pre, N = 23^1^	Post, N = 13^1^	Pre, N = 25^1^	Post, N = 21^1^
I am confident in my knowledge of neonatal resuscitation.	4^* ^(2,5)	4^* ^(3,5)	4^* ^(2,5)	4^* ^(3,5)	3^* ^(2,5)	4^* ^(3,5)	4 (2,5)	4 (3,5)
In my respective role, I am confident in neonatal airway management.	3^* ^(1,5)	4^* ^(2,5)	4 (1,5)	4 (2,5)	3^* ^(2,5)	4^* ^(3,5)	4^* ^(1,5)	4^* ^(2,5)
In my respective role, I am prepared to locate all equipment necessary and medications for a neonatal resuscitation.	4 (1,5)	4 (2,5)	4 (1,5)	4 (2,5)	3^* ^(1,5)	4^* ^(3,5)	4 (1,5)	4 (2,5)
I am confident in identifying high-risk deliveries and the need for resuscitation.	4 (1,5)	4 (1,5)	4 (1,5)	4 (2,5)	3 (1,5)	4 (1,5)	4 (3,5)	5 (3,5)
In my respective role, I am confident in my ability to manage medications needed in an emergency situation.	3 (1,5)	4 (1,5)	3 (1,5)	4 (2,5)	2 (1,5)	2 (1,5)	4 (2,5)	4 (2,5)
In my role, I am confident in my ability to perform emergency intravenous (IV) access.	2^* ^(1,5)	3^* ^(1,5)	3^* ^(1,4)	3^* ^(1,5)	1 (1,5)	1 (1,5)	3 (1,5)	4 (2,5)
In my respective role, I am confident in my ability to identify and manage neonatal respiratory distress.	4 (1,5)	4 (1,5)	4 (2,5)	4 (2,5)	3 (2,5)	4 (1,5)	4 (1,5)	4 (2,5)
In my role, I feel prepared to manage a neonatal code/resuscitation in the delivery room.	3^* ^(1,5)	4^* ^(1,5)	4 (1,5)	4 (2,5)	3 (1,5)	4 (1,5)	4 (1,5)	4 (2,5)
In my respective role, I am confident in my ability to manage a neonatal code/resuscitation in the operating room.	3^* ^(1,5)	4^* ^(1,5)	4 (1,5)	4 (2,5)	3 (1,5)	3 (1,5)	4 (1,5)	4 (1,5)
In my respective role, I am confident a leader will be identified during a neonatal resuscitation.	4 (1,5)	4 (1,5)	4 (2,5)	4 (3,5)	4 (1,5)	5 (1,5)	4 (2,5)	4 (1,5)
In my respective role, I feel confident that I have all the necessary support and additional resources necessary to successfully perform a neonatal resuscitation.	4^* ^ (1,5)	4^* ^ (2,5)	4 (1,5)	4 (3,5)	4 (1,5)	5 (2,5)	4 (2,5)	4 (3,5)
I feel confident that all my team members have the skills and knowledge to successfully perform a neonatal resuscitation.	4^* ^ (1,5)	4^* ^ (2,5)	4^* ^(1,5)	4^* ^(3,5)	4^* ^(1,5)	5^* ^(3,5)	4 (2,5)	4 (3,5)
In my respective role, I am confident in my ability to effectively communicate with team members.	4 (2,5)	4 (1,5)	4 (3,5)	4 (3,5)	4 (2,5)	4 (3,5)	4 (3,5)	4 (3,5)
I feel confident that the delivery room team operates as a cohesive unit with clear communication.	4^* ^(2,5)	4^* ^(1,5)	4^* ^(2,5)	4^* ^(3,5)	4 (2,5)	4 (2,5)	4 (3,5)	4 (3,5)

**Figure 1:**
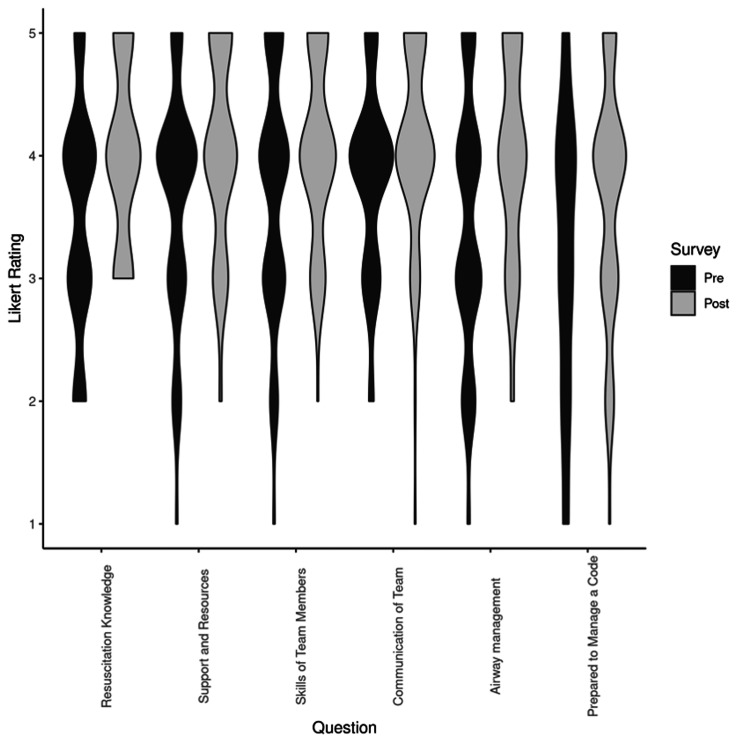
Confidence scores pre- and post-educational intervention as shown by violin plots. Six crucial areas of confidence were identified and shown in a violin plot (horizontal axis) compared with Likert scores (vertical axis).

In Figure 1, confidence scores for both the pre- and post-educational intervention are displayed as violin plots for six critical sub-categories: resuscitation knowledge, airway management, prepared to manage a code, support and resources, skills of team members, and team communication. Pre-survey responses are in black and post-survey responses are in grey. The violin plots demonstrate the improvement in the post-intervention confidence responses. In a limited sub-analysis by hospital, similar trends were observed with improvement in confidence scores post-simulation event. 

**Figure 2:**
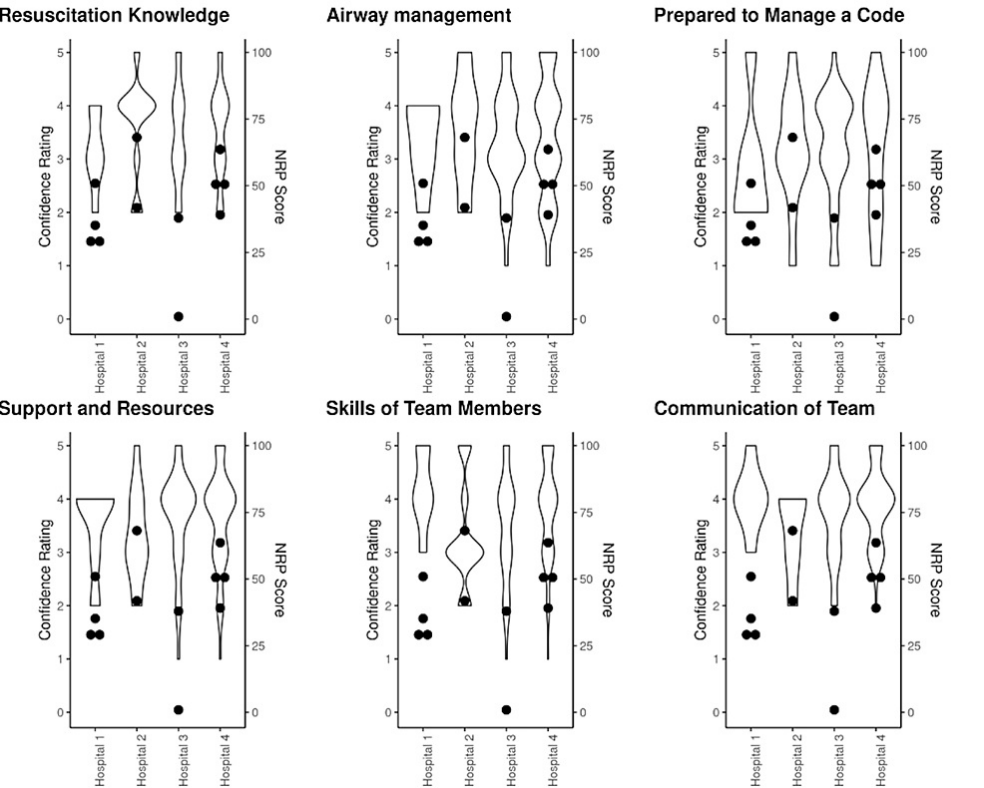
Confidence scores by question, prior to in situ event, compared with their baseline score performance (NRP® score) on simulation. Six crucial areas of confidence were identified and shown in a violin plot compared with NRP® scores (dots).  This demonstrates despite overall high confidence levels, NRP® scores remain low.

For the four hospitals with recorded simulated resuscitations, the NRP® adherence scores ranged widely from 1-68%. The one percent score reflected a lack of adherence to the current NRP® guidelines (examples include immediate intubation and suctioning for meconium and chest compressions performed before ventilation and airway were established). To compare confidence and NRP® adherence, we displayed pre-simulation confidence in Figure 2 as violin plots for six critical sub-categories as noted above. Figure 2 reveals a disconnect between high confidence and sub-optimal performance of NRP®, as measured by NRP® adherence. 

## Discussion

Our results demonstrate improved confidence in key aspects of neonatal resuscitation, teamwork, and communication after in situ simulation training in five rural community hospitals. Nurses and respiratory therapists showed the most improvement in their confidence, while the physician subgroup did not demonstrate significant changes. However, when we compare the resuscitation performance of the delivery room team as measured by NRP® adherence, we observe low adherence in the setting of relatively high confidence. While there is no established threshold for NRP® adherence, low adherence has the potential to adversely impact neonatal morbidity and mortality.

There are several potential explanations for the lack of concordance between confidence and the lower-than-expected NRP® adherence scores. It is possible that having participants review neonatal resuscitation materials prior to the event led to higher pre-event confidence and they had all completed NRP® training within the past two years. It is also possible that the novelty of the simulation environment for some participants led to poorer performance. Many participants had not previously experienced simulation training which involves suspending disbelief and engagement with the education modality. Team performance may also have been suboptimal as a result of the Hawthorne effect [14-17]; human behavior can improve or deteriorate when subjected to a higher-than-normal level of scrutiny. Lack of experience or engagement could have affected participants’ confidence levels.

The role (physician vs respiratory therapist vs nurse) and gender of the delivery room team member must also be considered when assessing confidence. In neonatal resuscitation, the physician is the leader of the resuscitation. In our study, the physicians had the highest pre- and post-simulation confidence (in other words had no change). Individuals in the physician group may have been involved with more high-risk resuscitations over their lifetimes and this may have influenced the lack of change in confidence. Increased experience is associated with higher baseline confidence in resuscitation [18] and prior experience with a medical procedure is associated with increased confidence in performing it [19]. Another factor for differences in confidence may be related to gender as multiple studies have shown women perceive themselves as less self-confident in many areas of medicine [20-22]. While responses in our survey were not linked to gender, ~50% of physician participants were female compared to 100% of nurse participants that were female. This may be related to the lower pre-assessment in confidence of the nurses. As neonatal resuscitation is a team-based, highly coordinated effort, improved confidence for any team member has the potential to benefit the performance of the whole group.

There are several strengths of this study including the study design, which provided participants with an intensive boot camp style skills training session with one-on-one instruction from neonatal care team experts prior to the simulation training. This allowed participants to build and refine resuscitation skills in a low-stakes situation with direct supervision that was not assessed or analyzed. Another strength is the in situ structure where the experts traveled to the rural community hospitals to provide training on location rather than having participants travel to the tertiary care center. This allowed participants to reflect on their own workflows (e.g., when to page the pediatrician) and equipment deficiencies (e.g., not having tape for the endotracheal tube in the intubation box), which could not have happened if the training was done at the tertiary care center.

There were also significant limitations to this study. To protect the anonymity of the participants, identifiers were not used. As a result, we were forced to use statistical tests for independent samples in our analysis and violate the independence assumption, which could have led to inaccurate conclusions. While all the delivery room team members who filled out the pre-training confidence survey took part in the training, there were 33 delivery room team members (32%), who did not complete the post-training survey. Without these data, we do not know if these individuals felt significantly more or less confident following training, and whether this had an impact on their decision to not fill out the post-survey. Furthermore, the majority of those that did not complete the post survey were respiratory therapists and APPs. It is unclear how this could affect the confidence data, but is worth addressing in future studies. Another limitation of our study is the lack of frequent simulation events at the same site over time for sustainability. While our results demonstrate improved confidence, multiple studies have demonstrated that resuscitation skills decline over time [23-30], often in as little as three months. In rural environments, frequent training sessions may be difficult to perform.

In the future, we plan on utilizing this data to generate further interventions that may help improve neonatal resuscitation. As one such intervention, we plan on trialing the use of telesimulation to allow more frequent simulation events and interactions with neonatal specialists.

## Conclusions

In conclusion, an in situ-based neonatal resuscitation outreach simulation program effectively improves self-confidence levels in many domains among delivery room teams. Additional research is needed to find ways to improve both self-confidence as well as NRP® adherence and to determine the ideal frequency of training. Our research team is currently investigating the use of telesimulation to improve the frequency of neonatal resuscitation simulations and to evaluate whether this correlates to an improvement in adherence to NRP® while preserving the benefit of in situ simulation in the rural hospital’s environment for continued assessment and improvement of systems.
